# Smoking and Risk of Erectile Dysfunction: Systematic Review of Observational Studies with Meta-Analysis

**DOI:** 10.1371/journal.pone.0060443

**Published:** 2013-04-03

**Authors:** Shiyi Cao, Xiaoxu Yin, Yunxia Wang, Hongfeng Zhou, Fujian Song, Zuxun Lu

**Affiliations:** 1 School of Public Health, Tongji Medical College, Huazhong University of Science and Technology, Wuhan, Hubei, China; 2 Norwich Medical School, Faculty of Medicine and Health Science, University of East Anglia, Norwich, United Kingdom; Tehran University of Medical Sciences, Iran (Islamic Republic of)

## Abstract

**Background:**

There are many recent observational studies on smoking and risk of erectile dysfunction (ED) and whether smoking increases the risk of ED is still inconclusive. The objective of this meta-analysis was to synthesize evidence from studies that evaluated the association between smoking and the risk of ED.

**Methods:**

We searched PubMed, Embase, Web of Science, and Scopus in January 2013 to identify cohort and case-control studies that evaluated the association between smoking and ED. Study quality of included studies was assessed by the Newcastle-Ottawa scale. Random-effects meta-analyses were used to combine the results of included studies.

**Results:**

Four prospective cohort studies and four case-control studies involving 28, 586 participants were included. Because of significant heterogeneity after including case-control studies in meta-analysis, the consistent results of prospective cohort studies were considered more accurate, Because of significant heterogeneity after including case-control studies in meta-analysis, the consistent results of prospective cohort studies were considered more accurate, Compared with non-smokers, the overall odd ratio of ED in prospective cohort studies was 1.51(95% CI: 1.34 to 1.71) for current smokers, and it was 1.29 (95% CI: 1.07 to 1.47) for former smokers. Evidence of publication bias was not found.

**Conclusion:**

Evidence from epidemiological studies suggests that smoking, especially current smoking, may significantly increase the risk of ED

## Introduction

Erectile dysfunction (ED), defined as the inability to attain and/or maintain penile erection [1], is common and increases as men age [2]. A number of risk factors are associated with ED, including psychological, neurological, endocrine, vascular, traumatic or iatrogenic causes [3]. Results of some epidemiological studies suggested that smoking, one of the world's greatest public health problems [4], may be related to the increased risk of ED in men.

In 2001, a meta-analysis [5] of 19 studies suggested a difference of 12.4% in the proportion of smokers between impotent men (40.1%) and general population (27.7%). Of note, in this meta-analysis the included studies were all conducted in the United State of America and the control group was drawn from the general population rather than from a group of men known to be free of ED. Additionally, majority of these studies were cross-sectional studies. We know that the strength of cross-sectional studies examining the association between a potential risk factor and a disease is very limited. In recent years, some cohort studies and case-control studies in various countries that examined the association between smoking and risk of ED have been published.

With accumulating evidence worldwide, we conducted a meta-analysis of cohort studies and case-control studies to evaluate the association between smoking and risk of ED in adult men.

## Methods

### Search strategy

We followed the Meta-Analysis of Observational Studies in Epidemiology [6] guidelines to report the present meta-analysis. We searched PubMed, Embase, Web of Science, and Scopus in January 2013 to identify cohort and case-control studies that investigated the association between smoking and risk of ED. The following search terms were used: (1) smoking, tobacco, or risk factors; and (2) erectile dysfunction, sexual dysfunction, or impotence. In addition, we checked the reference lists of retrieved papers and reviews. Search S1 shows the literature strategies used.

### Study selection

We first performed an initial screening of titles or abstracts to identify possibly relevant studies. Then we examined the full texts of studies identified based on titles and abstracts. Studies were considered eligible if they met the following criteria: (1) use of an cohort or case-control study design, (2) provision of sufficient data for calculating the association between smoking and ED in men aged older than 18, and (3) The ascertainment of ED was based on international index of erectile function (IIEF-5) Questionnaire or other self-designed questionnaires similar IIEF-5.

### Data extraction

For every eligible study, we collected detailed information on country or region of study, study design, age of study population, sample size, source of participants, definition or measurement of ED, confounders adjusted for, effect sizes, and 95% CIs or standard errors of effect sizes. Data were extracted independently by two of the investigators, and differences were resolved by discussion with a third author.

### Study quality assessment

We assessed the quality of all included studies by the Newcastle-Ottawa scale [7]. A quality score was calculated based on three major components: (1) selection of the groups of study, (2) comparability, (3) Assessment of the outcome or exposure. The maximum score could be 9 points, representing the highest methodological quality.

### Statistical analysis

Odds ratio (OR) was used as the common measure of the association between smoking and risk of ED across studies. For current smokers, or former smokers, we calculate the pooled ORs compared with never smokers. Former smokers are those who used to smoke before but don't smoke now.

Heterogeneity of ORs across studies was tested by using the Q statistic (significance level at *p*<0.10). The *I^2^* statistic, which is a quantitative measure of inconsistency across studies [8], was also calculated. We calculated an overall pooled OR using random effects model for the main analysis [9]. A *p* value<0.05 was considered statistically significant for the estimated ORs.

We conducted subgroup analyses to explore heterogeneity, across studies and the difference between subgroups was tested by meta-regression analysis (using STATA ‘metareg’ command) Potential publication bias was assessed by visual inspection of funnel plots in which the log ORs were plotted against their standard errors [10]. We also performed Egger' test of funnel plot asymmetry at the p<0.10 level of significance [11]. All analyses were performed using STATA version 11.0 (StataCorp LP, College Station Texas).

## Results

### Literature search

**Figure 1:**
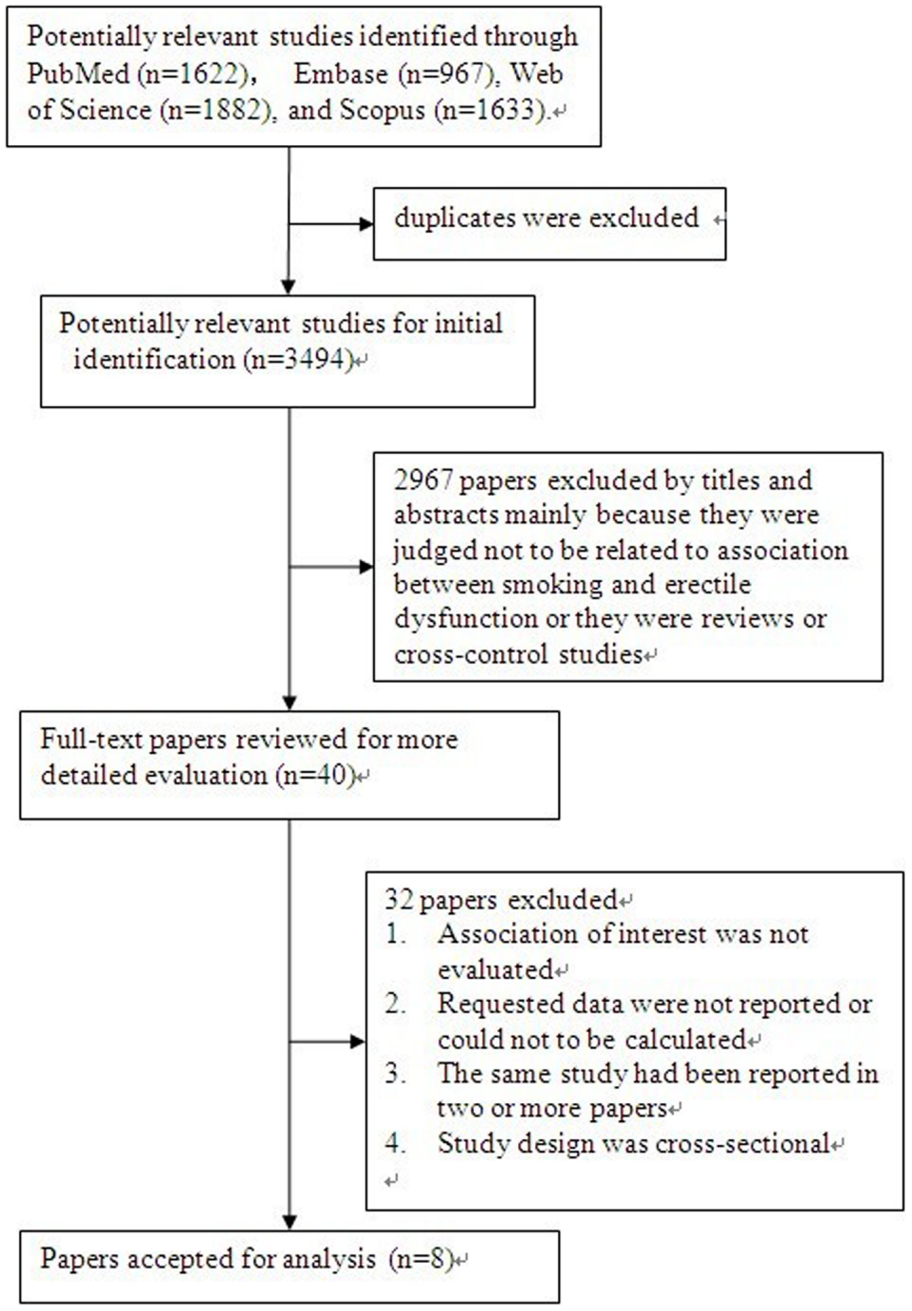
Flow Chart of Study Selection.

We initially retrieved 3494 unique citations from PubMed, Embase, Web of Science, and Scopus in January 2013. Of these, the majority were excluded after the first screening based on abstracts or titles, because they were not relevant or they were reviews or cross section studies. By examining the full-texts of 40 papers, we excluded 32 studies because association of interest was not evaluated, requested data were not reported, , of duplicate papers of the same studies, or study design was cross-sectional. Finally, eight studies were included in our meta-analysis [12]–[19]. Figure 1 shows the process of the study selection.

### Study characteristics

The main characteristics of the eight observational studies are presented in Table 1. These studies were published between 2000 and 2010. There were four case-control studies and four cohort studies. Of these studies, three were conducted in the United States, two in Egypt, one in Brazil, Jamaica, and Finland. Sample sizes ranged from 333 to 22,086 (total 28,586). Measurement of smoking was obtained by questionnaires in all the included studies. The ascertainment of ED varied across studies, with three based on international index of erectile function (IIEF-5) Questionnaire, one based on clinical diagnosis, one base on The Brief Male Sexual Function Inventory, and three based on other self-designed questionnaires. The risk of ED for current smokers was reported in all eight studies, and the risk of ED for former smokers was reported in only four of them. Estimation of ORs was not adjusted for confounding variables in one study. The number of confounding variables adjusted was only two (Hypertension and diabetes mellitus) in a study, and four or more variables were adjusted in the remaining studies. The quality of included studies was moderate or good, varying from five to eight points. (See Table S1).

**Table 1:** Characteristics of Included Studies.

Author (year)	Country or region	Type of study	Numbers of participants	Age of participants	source of participants	Assessment of ED	Adjustment for covariates
Bacon CG 2006	America	prospective cohort study (follow up 14 years)	22086	Range 40–75	Male health professionals	Self-designed questionnaire: Their ability (without treatment) to have and maintain an erection sufficient for intercourse was poor or very poor	Age, marital status, alcohol consumption, physical actively, obesity
Elbendary MA 2009	Egypt	Case-control study	706	younger than 40	men with ED and healthy volunteers	IIEF 5 Questionnaire	Recreation drugs, obesity, dyslipidemia, diabetes mellitus, hypertension, coronary heart disease, chronic pelvic pain syndrome
Feldman HA 2000	America	prospective cohort study (follow up 8.9 years)	513	Range 40–70	the general population	A privately self-administered and self-designed questionnaire	Passive cigarette exposure, cigar smoking, overweight, hypertension, alcohol consumption, moderate-heavy physical activity, serum cholesterol, high density lipoprotein cholesterol, serum dehydroepiandrosterone sulfate, saturated fat intake, unsaturated fat intake, dietary cholesterol, dietary fiber, anger index, age, serum testosterone, antihypertensive medication, depression
Gades NM 2005	America	prospective cohort study (follow up 14 years)	2115	Range 40–79	men who have the natural history of urinary symptoms and benign prostatic hyperplasia	The Brief Male Sexual Function Inventory	Age, the occurrence of hypertension, diabetes, or coronary heart disease
Polsky JY 2005	Jamaica	Case-control study	335	Range 50–80	men who visited one of a group of five urologists for various conditions	Clinically diagnosed ED	Age, alcohol intake, diabetes history, education level, and cardiovascular disease medications
Shiri R 2005	Finland	prospective ohort study (follow up 10 years)	1130	Rang 50–75	the general population	Positive answer about “have you had problems getting an erection before intercourse begins” or “have you had problems maintaining an erection once intercourse has begun”	Age, education, marital status and alcohol consumption
Zambon JP 2010	Brazil	Case-control study	222	Range 39–73	men who enrolled in a health review program	IIEF 5 Questionnaire	No covariates or not reported
Zedan H 2010	Egypt	Case-control study	1479	older than 20	men who attended a andrology clinic	IIEF 5 Questionnaire	Hypertension and diabetes mellitus

Abbreviations: ED: Erectile dysfunction. IIEF 5 Questionnaire: International Index of Erectile Function (5 Items) Questionnaire.

### Main results of meta-analysis

#### Current smoking and risk of ED

**Figure 2:**
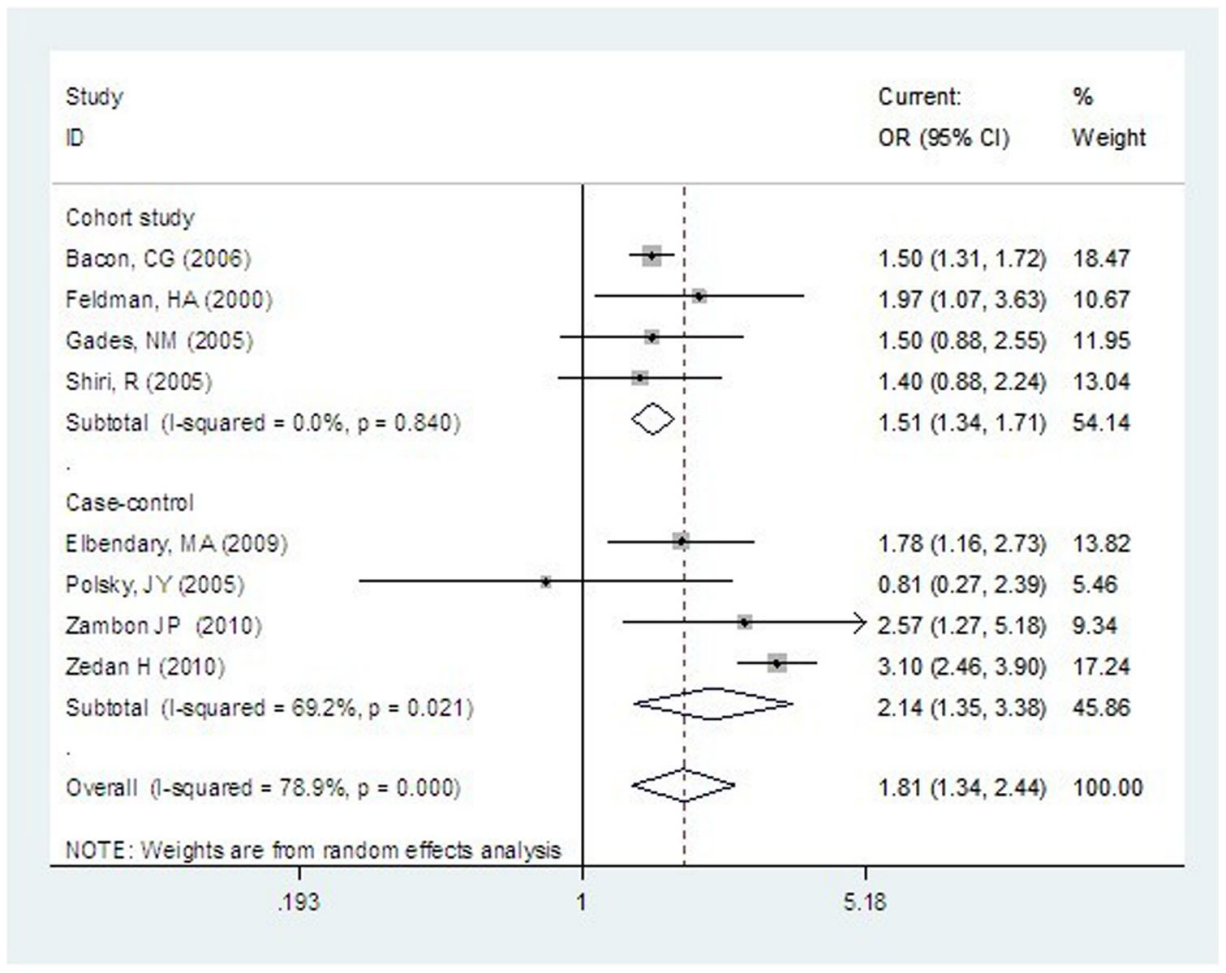
Current Smoking and Risk of ED.

Two of the four cohort studies and three of the four case-control studies reported statistically significant association between current smoking and the risk of ED (Figure 2). The combined OR was 1.15 (95% CI: 1.34 to 1.71) for prospective cohort studies, and it was 2.14 (95% CI: 1.35 to 3.38) for case-control studies. Heterogeneity in results was not significant across prospective cohort studies (I^2^ = 0.0%; P = 0.84), but it was statistically significant across case-control studies (I^2^ = 69.2%; P = 0.021).

#### Ex-smoking and risk of ED

**Figure 3:**
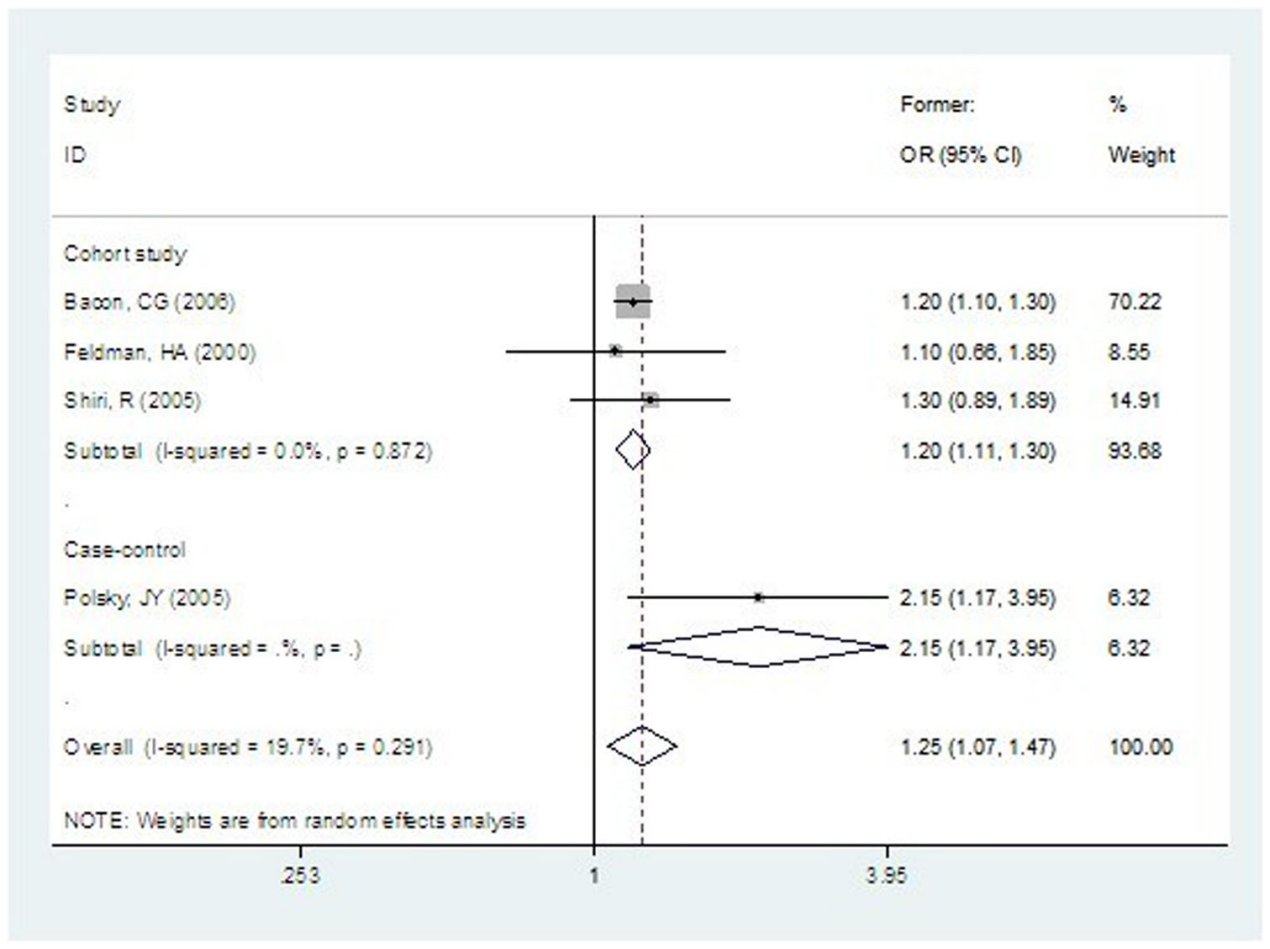
Ex-smoking and Risk of ED.

Based on data from three prospective cohort studies, the combined OR for ex-smokers was 1.20 (95% CI: 1.11 to 1.30), and there was no significant heterogeneity across studies (Figure 3). The association between ex-smoking and ED was reported in only one of the four case-control studies.

### Results of Subgroup analysis and sensitivity analyses

Subgroup analysis by study design showed that ORs estimated by cohort studies tended to be smaller and more consistent than the results of case-control studies (Figure 2 and Figure 3). For the association between current smoking and the risk of ED, the difference in results between cohort studies and case-control studies was considerable (P = 0.092).

A meta-regression analysis found that the number of covariates adjusted in analysis was not associated with the estimated OR (P = 0.623). However, subgroup analysis based on adjustment of confounders showed that the pooled OR for studies with adjustment for two or less covariates was bigger than that for studies with adjustment for three or more important covariates (3.04[95% CI: 2.45 to 3.79] vs. 1.52[95% CI: 1.35 to 1.71]). These two subgroup both did not show substantial heterogeneity (*p* = 0.534, *I^2^* = 0.0% and *p* = 0.572, *I^2^* = 0.0%). The difference in results between the two subgroups was statistically significant (P = 0.017).

Subgroup analysis based on the assessment method of ED suggested that the pooled OR for studies that assessed ED using IIEF 5 Questionnaire, the Brief Male Sexual Function Inventory, or clinical diagnosis was 1.98 (95% CI: 1.31 to 2.99) with substantial heterogeneity (*p* = 0.009, *I^2^* = 70.3%) and the pooled OR for studies that assessed ED based on self-design questionnaire was 1.51 (95% CI: 1.33 to 1.71) without heterogeneity (*p* = 0.658, *I^2^* = 0.0%).

In a univariate meta-regression analysis, it was found that a higher quality score was associated with a smaller OR (P = 0.051).

### Publication bias

Visual inspection of the funnel plot did not identify substantial asymmetry (see Figure S1). The test of funnel plot asymmetry indicated no evidence of publication bias among studies of current smoking and ED risk (Egger' test *P* = 0.866).

## Discussion

This systematic review included four perspective cohort studies and four case-control studies involving 28,842 participants. In this meta-analysis, the results of the included case-control studies were significantly heterogeneous and may have over-estimated the association between smoking and ED. Therefore, we consider the consistent results of prospective studies are more accurate. The results from prospective cohort studies suggested that the risk of ED was increased by 51% for current smokers and 20% for ex-smokers, as compared with never-smokers. The results also suggested that the increased risk of ED associated with smoking may decrease after stopping smoking.

There were considerable differences in characteristics of populations, study design, ascertainment of ED, and adjustment for confounding factors (see Table 1). Heterogeneity is often a concern in meta-analysis. For analyzing the association between current smoking and ED, heterogeneity was statistically significant when cohort studies and case-control studies were combined, although heterogeneity was non-significant across prospective cohort studies (Figure 2). In addition, the results of subgroup analyses suggested that the number of covariates adjusted and study quality may also be important variables associated with heterogeneity across studies.

The present study has the following limitations. First, we did not include cross-sectional studies that reported association between smoking and ED, due to resource limitations and the methodological weaknesses of the design. Secondly, methods used to diagnose ED were different across the included studies. Only three of the included studies usedIIEF-5 questionnaire, although it is an internationally recognized tool to diagnose ED. Some studies assessed ED by clinical diagnosis or other self-design questionnaire. Third, possible confounding effects were adjusted differently in the included studies, and it was unclear about what covariates should be adjusted in analyses and whether the adjusted ORs were actually more valid than the unadjusted estimates.

In summary, it may be concluded that the risk of ED is higher in current and former smokers than never smokers, although smoking cessation may be associated with a lower risk of ED than current smoking.

## Supporting Information

Search S1 **Literature search strategy.**
(DOC)

Table S1 **Study quality of included studies based on the Newcastle-Ottawa scale.**
(DOC)

Figure S1 **Funnel plot of the meta-analyses of current smoking and risk of ED.**
(TIF)

## References

[1] (1993) NIH Consensus Conference. Impotence. NIH Consensus Development Panel on Impotence. JAMA 270: 83–90. 8510302

[2] Dong JY, Zhang YH, Qin LQ (2011) Erectile dysfunction and risk of cardiovascular disease: meta-analysis of prospective cohort studies. J Am Coll Cardiol 58: 1378–1385. 21920268 10.1016/j.jacc.2011.06.024

[3] Kirby RS (1994) Impotence: diagnosis and management of male erectile dysfunction. BMJ 308: 957–961. 8173405 10.1136/bmj.308.6934.957 PMC2539778

[4] Bates MN, Khalakdina A, Pai M, Chang L, Lessa F, et al (2007) Risk of tuberculosis from exposure to tobacco smoke: a systematic review and meta-analysis. Arch Intern Med 167: 335–342. 17325294 10.1001/archinte.167.4.335

[5] Tengs TO, Osgood ND (2001) The link between smoking and impotence: two decades of evidence. Prev Med 32: 447–452. 11394947 10.1006/pmed.2001.0830

[6] Stroup DF, Berlin JA, Morton SC, Olkin I, Williamson GD, et al (2000) Meta-analysis of observational studies in epidemiology: a proposal for reporting. Meta-analysis Of Observational Studies in Epidemiology (MOOSE) group. JAMA 283: 2008–2012. 10789670 10.1001/jama.283.15.2008

[7] Wells G, Shea B, O'Connell D (2010) The Newcastle–Ottawa Scale (NOS) for assessing the quality of nonrandomised studies in meta-analyses. Ottawa (ON): Ottawa Health Research Institute.

[8] Higgins JP, Thompson SG, Deeks JJ, Altman DG (2003) Measuring inconsistency in meta-analyses. BMJ 327: 557–560. 12958120 10.1136/bmj.327.7414.557 PMC192859

[9] DerSimonian R, Laird N (1986) Meta-analysis in clinical trials. Control Clin Trials 7: 177–188. 3802833 10.1016/0197-2456(86)90046-2

[10] Begg CB, Mazumdar M (1994) Operating characteristics of a rank correlation test for publication bias. Biometrics 50: 1088–1101. 7786990

[11] Peters JL, Sutton AJ, Jones DR, Abrams KR, Rushton L (2006) Comparison of two methods to detect publication bias in meta-analysis. JAMA 295: 676–680. 16467236 10.1001/jama.295.6.676

[12] Bacon CG, Mittleman MA, Kawachi I, Giovannucci E, Glasser DB, et al (2006) A prospective study of risk factors for erectile dysfunction. J Urol 176: 217–221. 16753404 10.1016/S0022-5347(06)00589-1

[13] Elbendary MA, El-Gamal OM, Salem KA (2009) Analysis of risk factors for organic erectile dysfunction in Egyptian patients under the age of 40 years. J Androl 30: 520–524. 19234310 10.2164/jandrol.108.007195

[14] Feldman HA, Johannes CB, Derby CA, Kleinman KP, Mohr BA, et al (2000) Erectile dysfunction and coronary risk factors: prospective results from the Massachusetts male aging study. Prev Med 30: 328–338. 10731462 10.1006/pmed.2000.0643

[15] Gades NM, Nehra A, Jacobson DJ, McGree ME, Girman CJ, et al (2005) Association between smoking and erectile dysfunction: a population-based study. Am J Epidemiol 161: 346–351. 15692078 10.1093/aje/kwi052

[16] Polsky JY, Aronson KJ, Heaton JP, Adams MA (2005) Smoking and other lifestyle factors in relation to erectile dysfunction. BJU Int 96: 1355–1359. 16287457 10.1111/j.1464-410X.2005.05820.x

[17] Shiri R, Hakama M, Hakkinen J, Tammela TL, Auvinen A, et al (2005) Relationship between smoking and erectile dysfunction. Int J Impot Res 17: 164–169. 15510179 10.1038/sj.ijir.3901280

[18] Zambon JP, Mendonca RR, Wroclawski ML, Karam Junior A, Santos RD, et al (2010) Cardiovascular and metabolic syndrome risk among men with and without erectile dysfunction: case-control study. Sao Paulo Med J 128: 137–140. 20963365 10.1590/S1516-31802010000300006 PMC10938961

[19] Zedan H, Hareadei AA, Abd-Elsayed AA, Abdel-Maguid EM (2010) Cigarette smoking, hypertension and diabetes mellitus as risk factors for erectile dysfunction in upper Egypt. East Mediterr Health J 16: 281–285. 20795441

