# Supplementary material for: Smoking and Risk of Erectile Dysfunction: Systematic Review of Observational Studies with Meta-Analysis
Source: PLoS One. 2013 Apr 3;8(4):e60443. doi: 10.1371/journal.pone.0060443 (PMC3616119; doi:10.1371/journal.pone.0060443)
Supplement: Search S1 — Literature search strategy. (DOC) [file pone.0060443.s001.doc]

# Literature search strategy

1. PubMed was searched using the following terms：

(erectile dysfunction OR sexual dysfunction OR impotence) AND (smok* OR tobacco OR risk factors)

Limits Activated: Humans, Male, All Adult: 19+ years

1. Embase was searched with the following terms:

'erectile dysfunction'/exp OR 'sexual dysfunction'/exp OR 'impotence'/exp AND (smok* OR 'tobacco'/exp OR 'risk factors'/exp) AND [male]/lim AND ([adult]/lim OR [aged]/lim) AND [humans]/lim

1. Web of Science was searched with the following terms:

Topic=(“Erectile dysfunction” or “sexual dysfunction” or impotence) AND Topic= (smok* or “risk factors”)

1. Scopus was searched with the following terms:

ALL(“Erectile dysfunction” or “sexual dysfunction” or impotence) AND ALL (smok* or “risk factors”)

1. References of retrieved articles will also be examined to identify relevant studies.
