# Supplementary material for: Smoking and Risk of Erectile Dysfunction: Systematic Review of Observational Studies with Meta-Analysis
Source: PLoS One. 2013 Apr 3;8(4):e60443. doi: 10.1371/journal.pone.0060443 (PMC3616119; doi:10.1371/journal.pone.0060443)
Supplement: Table S1 — Study quality of included studies based on the Newcastle-Ottawa scale. (DOC) [file pone.0060443.s002.doc]

# Study quality of included studies based on the Newcastle-Ottawa scale

## 3.1 Study quality of case-control studies

| **Author** | **Is the case definition adequate?** | **Representativeness of the Cases** | **Selection of Controls** | **Definition of Controls** | **Comparability of Cases and Controls on the Basis of the Design or Analysis** | **Ascertainment of exposure** | **Same method of ascertainment for cases and controls** | **Non-Response rate** | **Total scores** |
| --- | --- | --- | --- | --- | --- | --- | --- | --- | --- |
| Elbendary MA 2009 | ★ | ★ | ★ | ★ | ☆☆ | ★ | ★ | ☆ | 6 |
| Polsky JY 2005 | ★ | ★ | ☆ | ★ | ★★ | ★ | ★ | ☆ | 7 |
| Zambon JP 2010 | ★ | ★ | ★ | ★ | ☆☆ | ★ | ★ | ☆ | 6 |
| [Zedan H](http://www.ncbi.nlm.nih.gov/pubmed?term="Zedan H"%5BAuthor%5D) 2010 | ★ | ★ | ☆ | ★ | ☆☆ | ★ | ★ | ☆ | 5 |

## 3.2 Study quality of cohort studies

| **Author** | **Representativeness of the exposed cohort** | **Selection of the non exposed cohort** | **Ascertainment of exposure** | **Demonstration that outcome of interest was not present at start of study** | **Comparability of cohorts on the basis of the design or analysis** | **Assessment of outcome** | **Was follow-up long enough for outcomes to occur** | **Adequacy of follow up of cohorts** | **Total scores** |
| --- | --- | --- | --- | --- | --- | --- | --- | --- | --- |
| Bacon CG 2006 | ☆ | ★ | ★ | ★ | ★★ | ☆ | ★ | ★ | 8 |
| Feldman HA 2000 | ★ | ★ | ★ | ★ | ★★ | ☆ | ☆ | ☆ | 6 |
| Gades NM 2005 | ☆ | ★ | ★ | ★ | ★★ | ★ | ★ | ★ | 7 |
| Shiri R 2005 | ★ | ★ | ★ | ★ | ★★ | ☆ | ☆ | ☆ | 6 |
