# Supplementary figures and images for: Smoking and Risk of Erectile Dysfunction: Systematic Review of Observational Studies with Meta-Analysis
Source: PLoS One. 2013 Apr 3;8(4):e60443. doi: 10.1371/journal.pone.0060443 (PMC3616119; doi:10.1371/journal.pone.0060443)

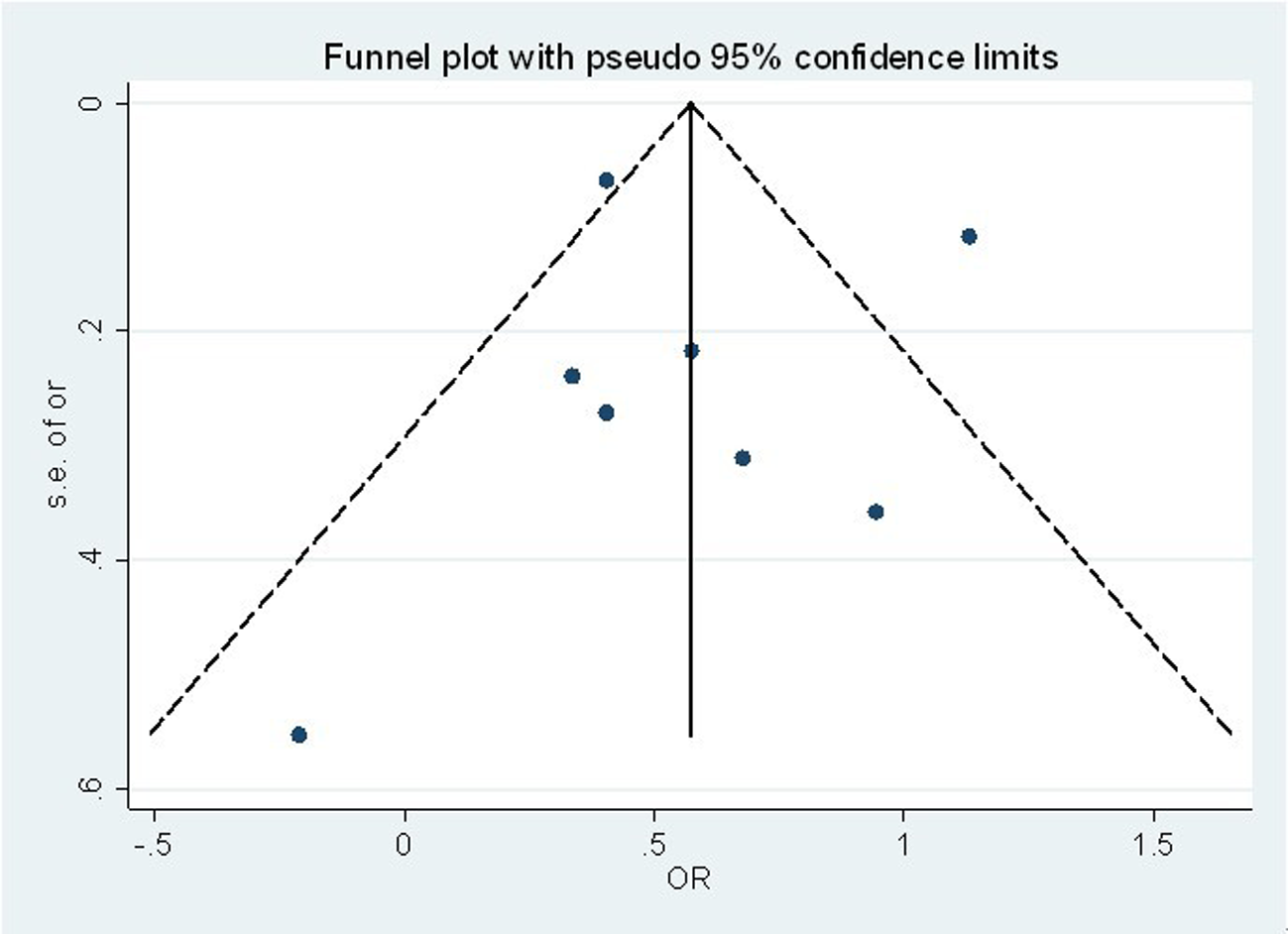

Supplement: Figure S1 — Funnel plot of the meta-analyses of current smoking and risk of ED. (TIF) [file pone.0060443.s003.tif]
